# 1-[(2-Chloro-8-methyl­quinolin-3-yl)­meth­yl]pyridin-2(1*H*)-one

**DOI:** 10.1107/S1600536810012730

**Published:** 2010-04-10

**Authors:** Atul Kumar Kushwaha, S. Mohana Roopan, F. Nawaz Khan, Venkatesha R. Hathwar, Mehmet Akkurt

**Affiliations:** a Organic and Medicinal Chemistry Research Laboratory, Organic Chemistry Division, School of Advanced Sciences, VIT University, Vellore 632 014, Tamil Nadu, India; b Solid State and Structural Chemistry Unit, Indian Institute of Science, Bangalore 560 012, Karnataka, India; c Department of Physics, Faculty of Arts and Sciences, Erciyes University, 38039 Kayseri, Turkey

## Abstract

In the title compound, C_16_H_13_ClN_2_O, the quinoline ring system is approximately planar [maximum deviation 0.021 (2) Å] and forms a dihedral angle of 85.93 (6)° with the pyridone ring. Inter­molecular C—H⋯O hydrogen bonding, together with weak C—H⋯π and π–π inter­actions [centroid-to-centroid distances 3.5533 (9) and 3.7793 (9) Å], characterize the crystal structure.

## Related literature

For 2-pyridone analogues, see: Arman *et al.* (2009 ▶); Clegg & Nichol (2004 ▶); Nichol & Clegg (2005 ▶). For the synthesis of 2-pyridone derivatives, see: Banerjee & Sereda (2009 ▶); Roopan & Khan (2009 ▶); Roopan *et al.* (2010 ▶); Dandepally & Williams (2009 ▶).
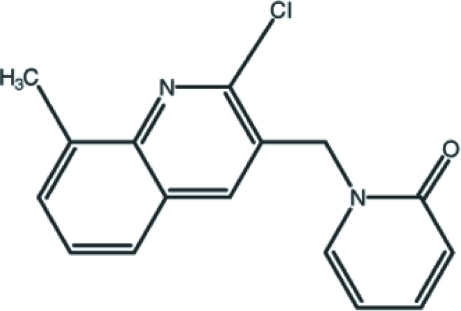


## Experimental

### 

#### Crystal data

C_16_H_13_ClN_2_O
*M* *_r_* = 284.73
Monoclinic, 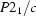

*a* = 10.1513 (2) Å
*b* = 9.3917 (2) Å
*c* = 14.1430 (2) Å
β = 90.948 (2)°
*V* = 1348.17 (4) Å^3^
*Z* = 4
Mo *K*α radiation
μ = 0.28 mm^−1^
*T* = 295 K
0.26 × 0.24 × 0.20 mm

#### Data collection

Oxford Xcalibur Eos (Nova) CCD detector diffractometer
Absorption correction: multi-scan (*CrysAlis PRO*; Oxford Diffraction, 2009 ▶) *T*_min_ = 0.931, *T*_max_ = 0.946
17649 measured reflections
2511 independent reflections
2088 reflections with *I* > 2σ(*I*)
*R*_int_ = 0.033

#### Refinement

*R*[*F* ^2^ > 2σ(*F* ^2^)] = 0.034
*wR*(*F* ^2^) = 0.100
*S* = 1.10
2511 reflections
182 parameters
H-atom parameters constrained
Δρ_max_ = 0.16 e Å^−3^
Δρ_min_ = −0.33 e Å^−3^

### 

Data collection: *CrysAlis PRO* (Oxford Diffraction, 2009 ▶); cell refinement: *CrysAlis PRO*; data reduction: *CrysAlis PRO*; program(s) used to solve structure: *SHELXS97* (Sheldrick, 2008 ▶); program(s) used to refine structure: *SHELXL97* (Sheldrick, 2008 ▶); molecular graphics: *ORTEP-3 for Windows* (Farrugia, 1997 ▶); software used to prepare material for publication: *WinGX* (Farrugia, 1999 ▶) and *PLATON* (Spek, 2009 ▶).

## Supplementary Material

Crystal structure: contains datablocks global, I. DOI: 10.1107/S1600536810012730/im2191sup1.cif

Structure factors: contains datablocks I. DOI: 10.1107/S1600536810012730/im2191Isup2.hkl

Additional supplementary materials:  crystallographic information; 3D view; checkCIF report

## Figures and Tables

**Table 1:** Hydrogen-bond geometry (Å, °) *Cg*1 is the centroid of the N1/C1–C3/C8/C9 ring.

*D*—H⋯*A*	*D*—H	H⋯*A*	*D*⋯*A*	*D*—H⋯*A*
C11—H11⋯O1^i^	0.93	2.54	3.286 (2)	137
C6—H6⋯*Cg*1^ii^	0.93	2.61	3.4457 (18)	150

Symmetry codes: (i) 
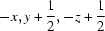
; (ii) 
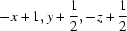
.

**Fig. 1.**
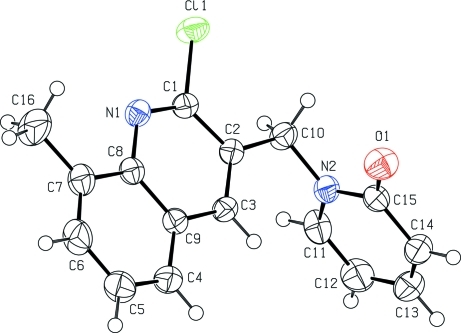
The title molecule with the atom numbering scheme. Displacement ellipsoids for non-H atoms are drawn at the 50% probability level.

**Fig. 2.**
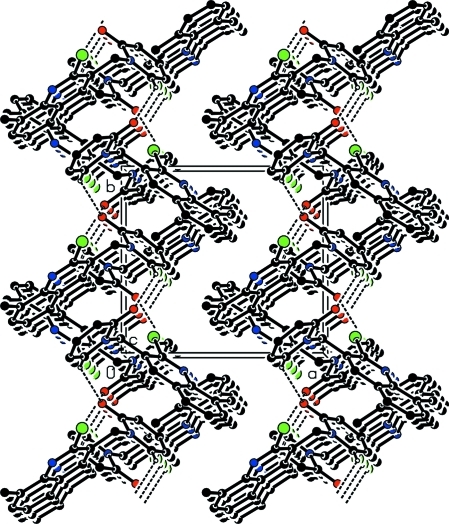
The packing diagram and the hydrogen bonding interactions of the title compound viewed down c axis. H atoms not involved in hydrogen bonding have been omitted for clarity.

## supplementary crystallographic information

### Comment

As part of our search for new quinoline analogues, we focused on *N*-alkylation of 2-pyridinone using 2-chloro-3-(chloromethyl)-8-methylquinoline. *N*-alkylations are used in the synthesis of various heterocyclic (Dandepally & Williams, 2009) naturally occurring alkaloids. The chemistry of *N*-alkylation has received much attention due to their usefulness as building blocks in organic synthesis (Roopan *et al.*, 2010). Compounds found in nature display a wide range of diversity in terms of their structures and physical and biological properties. The synthesis of privileged medicinal scaffolds is highly important as these compounds often act as a platform for developing pharmaceutical agents for diverse applications (Roopan & Khan, 2009). These vast applications have inspired the development of a number of methods for the preparation of pyridine nucleus (Banerjee & Sereda, 2009). However, literature studies reveal that most of the methods involve low isolated yields and long reaction times. On the basis of the interesting structures and biological activities exhibited by several heterocyclic systems possessing quinoline and pyridinone nuclei, we have synthesized a quinoline coupled pyridinone, i.e. 1-[(2-chloro-8-methylquinolin-3yl)-methyl]-pyridine-2(1*H*)-one.

The quinoline ring system (N1/C1–C3/C8/C9) of the title molecule in Fig. 1 is approximately planar, with maximum deviations of 0.021 (2) Å for C7, -0.021 (1) Å for N1 and 0.018 (2) Å for C5. It makes a dihedral angle of 85.93 (6)° with the pyridinone ring (N2/C11–C15). Intramolecular C—H···N, intermolecular C—H···O hydrogen bonding, together with weak C—H···π (Table 1) and π–π interactions [*Cg*1···*Cg*2(-*x*, 1/2 + *y*, 1/2 - *z*) = 3.5533 (9) Å and *Cg*2···*Cg*3(-*x*, -1/2 + *y*, 1/2 - *z*) = 3.7793 (9) Å, where *Cg*1, *Cg*2 and *Cg*3 are the centroids of the N1/C1–C3/C8/C9, N2/C11–C15 and C4–C9 rings, respectively], characterize the crystal structure. Fig. 2 shows the hydrogen bonding in terms of a packing diagrams of the title compound.

### Experimental

To a vigorously stirred solution of 2-pyridinone (95 mg, 1 mmol, in 2 ml DMF) KO^t^Bu (112 mg, 1 mmol, in 10 ml THF) and 2-chloro-3-(chloromethyl)-8-methylquinoline (226 mg, 1 mmol) were added and the resulting mixture was refluxed at 343 K for 1 h. After the completion of the reaction it was cooled to room temperature and the excess of solvent was removed under reduced pressure. Crushed ice was mixed with the residue producing a white solid that was filtered and dried. Purification was performed by column chromatography using hexane and ethyl acetate (1:9) as the eluant. Crystals of suitable quality were grown by solvent evaporation from a solution of the compound in dichloromethane at room temperature.

### Refinement

H atoms were located geometrically with C—H = 0.93–0.97 Å and refined using a riding model, with U_iso_(H) = 1.5U_eq_(C) for methyl H and U_iso_(H) = 1.2U_eq_(C) for all other H atoms.

### Crystal data

**Table:**

C_16_H_13_ClN_2_O	*F*(000) = 592
*M**_r_* = 284.73	*D*_x_ = 1.403 Mg m^−^^3^
Monoclinic, *P*2_1_/*c*	Mo *K*α radiation, λ = 0.71073 Å
Hall symbol: -P 2ybc	Cell parameters from 1116 reflections
*a* = 10.1513 (2) Å	θ = 2.0–21.0°
*b* = 9.3917 (2) Å	µ = 0.28 mm^−^^1^
*c* = 14.1430 (2) Å	*T* = 295 K
β = 90.948 (2)°	Block, colourless
*V* = 1348.17 (4) Å^3^	0.26 × 0.24 × 0.20 mm
*Z* = 4	

### Data collection

**Table:**

Oxford Xcalibur Eos (Nova) CCD detector diffractometer	2511 independent reflections
Radiation source: Enhance (Mo) X-ray Source	2088 reflections with *I* > 2σ(*I*)
graphite	*R*_int_ = 0.033
ω scans	θ_max_ = 25.5°, θ_min_ = 3.0°
Absorption correction: multi-scan (*CrysAlis PRO*; Oxford Diffraction, 2009)	*h* = −12→12
*T*_min_ = 0.931, *T*_max_ = 0.946	*k* = −11→11
17649 measured reflections	*l* = −17→17

### Refinement

**Table:**

Refinement on *F*^2^	Primary atom site location: structure-invariant direct methods
Least-squares matrix: full	Secondary atom site location: difference Fourier map
*R*[*F*^2^ > 2σ(*F*^2^)] = 0.034	Hydrogen site location: inferred from neighbouring sites
*wR*(*F*^2^) = 0.100	H-atom parameters constrained
*S* = 1.10	*w* = 1/[σ^2^(*F*_o_^2^) + (0.0626*P*)^2^ + 0.062*P*] where *P* = (*F*_o_^2^ + 2*F*_c_^2^)/3
2511 reflections	(Δ/σ)_max_ < 0.001
182 parameters	Δρ_max_ = 0.16 e Å^−^^3^
0 restraints	Δρ_min_ = −0.33 e Å^−^^3^

### Special details

**Table:**

Geometry. Bond distances, angles etc. have been calculated using the rounded fractional coordinates. All su's are estimated from the variances of the (full) variance-covariance matrix. The cell esds are taken into account in the estimation of distances, angles and torsion angles
Refinement. Refinement on *F*^2^ for ALL reflections except those flagged by the user for potential systematic errors. Weighted *R*-factors wR and all goodnesses of fit *S* are based on *F*^2^, conventional *R*-factors *R* are based on *F*, with *F* set to zero for negative *F*^2^. The observed criterion of *F*^2^ > σ(*F*^2^) is used only for calculating -*R*-factor-obs etc. and is not relevant to the choice of reflections for refinement. *R*-factors based on *F*^2^ are statistically about twice as large as those based on *F*, and *R*-factors based on ALL data will be even larger.

### Fractional atomic coordinates and isotropic or equivalent isotropic displacement parameters (Å^2^)

**Table:**

	*x*	*y*	*z*	*U*_iso_*/*U*_eq_	
Cl1	0.17214 (4)	0.42990 (4)	0.02479 (2)	0.0424 (1)	
O1	0.08041 (10)	0.28868 (13)	0.35614 (8)	0.0481 (4)	
N1	0.32267 (11)	0.61310 (13)	0.11096 (8)	0.0315 (4)	
N2	−0.05971 (11)	0.45168 (13)	0.28993 (8)	0.0331 (4)	
C1	0.21630 (13)	0.53822 (15)	0.12066 (9)	0.0292 (4)	
C2	0.13534 (13)	0.53361 (14)	0.20141 (9)	0.0281 (4)	
C3	0.17568 (13)	0.61625 (16)	0.27547 (10)	0.0304 (4)	
C4	0.33798 (15)	0.78326 (17)	0.34745 (11)	0.0407 (5)	
C5	0.45224 (16)	0.8575 (2)	0.33878 (12)	0.0498 (6)	
C6	0.52323 (16)	0.8519 (2)	0.25493 (12)	0.0499 (6)	
C7	0.48186 (15)	0.77503 (17)	0.17819 (12)	0.0405 (5)	
C8	0.36339 (13)	0.69521 (15)	0.18602 (10)	0.0307 (4)	
C9	0.29139 (13)	0.69933 (15)	0.27116 (10)	0.0305 (4)	
C10	0.01318 (15)	0.44264 (17)	0.20158 (10)	0.0365 (5)	
C11	−0.16498 (15)	0.54157 (17)	0.29568 (12)	0.0430 (5)	
C12	−0.23606 (17)	0.54973 (19)	0.37505 (14)	0.0520 (6)	
C13	−0.20074 (17)	0.4635 (2)	0.45186 (13)	0.0507 (6)	
C14	−0.09706 (16)	0.37454 (18)	0.44725 (11)	0.0433 (5)	
C15	−0.01782 (14)	0.36501 (16)	0.36452 (10)	0.0342 (5)	
C16	0.55794 (17)	0.7727 (2)	0.08775 (14)	0.0626 (7)	
H3	0.12600	0.61800	0.33010	0.0360*	
H4	0.29090	0.78780	0.40320	0.0490*	
H5	0.48340	0.91260	0.38910	0.0600*	
H6	0.60170	0.90260	0.25140	0.0600*	
H10A	−0.04430	0.47170	0.14960	0.0440*	
H10B	0.03800	0.34430	0.19090	0.0440*	
H11	−0.18800	0.59800	0.24400	0.0520*	
H12	−0.30730	0.61160	0.37870	0.0630*	
H13	−0.24950	0.46760	0.50690	0.0610*	
H14	−0.07620	0.31770	0.49920	0.0520*	
H16A	0.51020	0.82450	0.03970	0.0940*	
H16B	0.56960	0.67590	0.06760	0.0940*	
H16C	0.64250	0.81610	0.09820	0.0940*	

### Atomic displacement parameters (Å^2^)

**Table:**

	*U*^11^	*U*^22^	*U*^33^	*U*^12^	*U*^13^	*U*^23^
Cl1	0.0429 (2)	0.0526 (3)	0.0316 (2)	−0.0044 (2)	0.0012 (2)	−0.0104 (2)
O1	0.0446 (7)	0.0512 (7)	0.0485 (7)	0.0098 (6)	0.0006 (5)	0.0002 (5)
N1	0.0289 (6)	0.0345 (7)	0.0311 (6)	0.0019 (5)	0.0023 (5)	0.0027 (5)
N2	0.0289 (6)	0.0350 (7)	0.0354 (7)	−0.0063 (5)	0.0027 (5)	−0.0005 (5)
C1	0.0295 (7)	0.0312 (8)	0.0269 (7)	0.0038 (6)	−0.0021 (5)	0.0010 (6)
C2	0.0258 (7)	0.0286 (7)	0.0298 (7)	0.0023 (6)	0.0000 (5)	0.0022 (6)
C3	0.0297 (7)	0.0331 (8)	0.0286 (7)	0.0013 (6)	0.0038 (5)	−0.0001 (6)
C4	0.0464 (9)	0.0410 (9)	0.0347 (8)	−0.0080 (7)	0.0020 (7)	−0.0044 (7)
C5	0.0568 (11)	0.0489 (10)	0.0433 (9)	−0.0172 (9)	−0.0076 (8)	−0.0057 (8)
C6	0.0423 (9)	0.0503 (10)	0.0570 (10)	−0.0196 (8)	−0.0006 (8)	0.0013 (9)
C7	0.0345 (8)	0.0406 (9)	0.0466 (9)	−0.0065 (7)	0.0036 (6)	0.0031 (7)
C8	0.0293 (7)	0.0288 (8)	0.0341 (7)	0.0018 (6)	−0.0005 (6)	0.0026 (6)
C9	0.0314 (7)	0.0288 (8)	0.0314 (7)	−0.0001 (6)	−0.0001 (6)	0.0007 (6)
C10	0.0358 (8)	0.0439 (9)	0.0299 (7)	−0.0073 (7)	0.0017 (6)	−0.0022 (6)
C11	0.0366 (9)	0.0367 (9)	0.0556 (10)	−0.0018 (7)	0.0006 (7)	0.0073 (8)
C12	0.0406 (9)	0.0459 (11)	0.0701 (12)	0.0062 (8)	0.0151 (8)	−0.0027 (9)
C13	0.0518 (10)	0.0521 (11)	0.0487 (10)	−0.0047 (8)	0.0186 (8)	−0.0046 (8)
C14	0.0475 (9)	0.0463 (10)	0.0363 (8)	−0.0073 (8)	0.0046 (7)	0.0008 (7)
C15	0.0351 (8)	0.0324 (8)	0.0352 (8)	−0.0072 (7)	−0.0003 (6)	−0.0032 (6)
C16	0.0502 (11)	0.0774 (14)	0.0609 (12)	−0.0236 (10)	0.0197 (9)	−0.0053 (10)

### Geometric parameters (Å, °)

**Table:**

Cl1—C1	1.7476 (14)	C11—C12	1.347 (3)
O1—C15	1.2354 (18)	C12—C13	1.397 (3)
N1—C1	1.2978 (18)	C13—C14	1.346 (2)
N1—C8	1.3704 (18)	C14—C15	1.434 (2)
N2—C10	1.4651 (18)	C3—H3	0.9300
N2—C11	1.3653 (19)	C4—H4	0.9300
N2—C15	1.3934 (19)	C5—H5	0.9300
C1—C2	1.4187 (18)	C6—H6	0.9300
C2—C3	1.3612 (19)	C10—H10A	0.9700
C2—C10	1.506 (2)	C10—H10B	0.9700
C3—C9	1.4123 (19)	C11—H11	0.9300
C4—C5	1.361 (2)	C12—H12	0.9300
C4—C9	1.412 (2)	C13—H13	0.9300
C5—C6	1.399 (2)	C14—H14	0.9300
C6—C7	1.364 (2)	C16—H16A	0.9600
C7—C8	1.423 (2)	C16—H16B	0.9600
C7—C16	1.505 (3)	C16—H16C	0.9600
C8—C9	1.419 (2)		
			
Cl1···O1^i^	3.2706 (12)	C14···C14^ix^	3.401 (2)
Cl1···H10A	2.8700	C15···C3^iv^	3.441 (2)
Cl1···H10A^ii^	2.9200	C15···C2^iv^	3.456 (2)
Cl1···H16B^iii^	3.1100	C15···C3	3.331 (2)
Cl1···H10B	2.8500	C1···H6^vi^	2.8600
O1···C2	3.2298 (17)	C2···H6^vi^	3.0000
O1···C2^iv^	3.3375 (17)	C3···H6^vi^	3.0500
O1···C11^iv^	3.286 (2)	C3···H10B^vii^	3.0900
O1···Cl1^v^	3.2706 (12)	C8···H6^vi^	2.9100
O1···H10B	2.4300	C9···H6^vi^	3.0100
O1···H11^iv^	2.5400	C11···H3	3.0700
O1···H16C^vi^	2.8900	C15···H3	2.8400
N1···C14^vii^	3.448 (2)	H3···N2	2.5100
N1···C5^vi^	3.382 (2)	H3···C11	3.0700
N2···C9^iv^	3.4391 (18)	H3···C15	2.8400
N1···H16B	2.6600	H3···H4	2.5200
N1···H5^vi^	2.7200	H3···H14^ix^	2.5500
N1···H6^vi^	2.8700	H4···H3	2.5200
N1···H16A	2.9400	H5···N1^viii^	2.7200
N2···H3	2.5100	H6···H16C	2.3600
C1···C14^vii^	3.511 (2)	H6···N1^viii^	2.8700
C2···C15^vii^	3.456 (2)	H6···C1^viii^	2.8600
C2···O1	3.2298 (17)	H6···C2^viii^	3.0000
C2···O1^vii^	3.3375 (17)	H6···C3^viii^	3.0500
C3···C15^vii^	3.441 (2)	H6···C8^viii^	2.9100
C3···C11	3.545 (2)	H6···C9^viii^	3.0100
C3···C15	3.331 (2)	H10A···Cl1	2.8700
C4···C11^vii^	3.599 (2)	H10A···H11	2.3200
C5···N1^viii^	3.382 (2)	H10A···Cl1^ii^	2.9200
C6···C8^viii^	3.519 (2)	H10B···Cl1	2.8500
C8···C13^vii^	3.574 (2)	H10B···O1	2.4300
C8···C6^vi^	3.519 (2)	H10B···C3^iv^	3.0900
C9···C11^vii^	3.582 (2)	H11···H10A	2.3200
C9···N2^vii^	3.4391 (18)	H11···O1^vii^	2.5400
C11···C9^iv^	3.582 (2)	H14···H3^ix^	2.5500
C11···C3	3.545 (2)	H16A···N1	2.9400
C11···C4^iv^	3.599 (2)	H16B···N1	2.6600
C11···O1^vii^	3.286 (2)	H16B···Cl1^iii^	3.1100
C13···C8^iv^	3.574 (2)	H16C···H6	2.3600
C14···N1^iv^	3.448 (2)	H16C···O1^viii^	2.8900
C14···C1^iv^	3.511 (2)		
			
C1—N1—C8	117.64 (12)	O1—C15—C14	125.40 (14)
C10—N2—C11	119.57 (12)	N2—C15—C14	114.40 (13)
C10—N2—C15	117.37 (12)	C2—C3—H3	119.00
C11—N2—C15	123.06 (12)	C9—C3—H3	119.00
Cl1—C1—N1	115.89 (10)	C5—C4—H4	120.00
Cl1—C1—C2	117.53 (10)	C9—C4—H4	120.00
N1—C1—C2	126.57 (12)	C4—C5—H5	120.00
C1—C2—C3	115.54 (12)	C6—C5—H5	120.00
C1—C2—C10	120.44 (12)	C5—C6—H6	119.00
C3—C2—C10	124.02 (12)	C7—C6—H6	119.00
C2—C3—C9	121.44 (13)	N2—C10—H10A	109.00
C5—C4—C9	119.52 (14)	N2—C10—H10B	109.00
C4—C5—C6	120.60 (16)	C2—C10—H10A	109.00
C5—C6—C7	122.57 (16)	C2—C10—H10B	109.00
C6—C7—C8	117.80 (15)	H10A—C10—H10B	108.00
C6—C7—C16	121.89 (15)	N2—C11—H11	120.00
C8—C7—C16	120.31 (14)	C12—C11—H11	119.00
N1—C8—C7	118.73 (13)	C11—C12—H12	121.00
N1—C8—C9	121.30 (12)	C13—C12—H12	121.00
C7—C8—C9	119.96 (13)	C12—C13—H13	120.00
C3—C9—C4	122.95 (13)	C14—C13—H13	120.00
C3—C9—C8	117.51 (13)	C13—C14—H14	119.00
C4—C9—C8	119.54 (13)	C15—C14—H14	119.00
N2—C10—C2	113.33 (12)	C7—C16—H16A	110.00
N2—C11—C12	121.03 (15)	C7—C16—H16B	109.00
C11—C12—C13	118.75 (16)	C7—C16—H16C	109.00
C12—C13—C14	120.86 (16)	H16A—C16—H16B	109.00
C13—C14—C15	121.87 (15)	H16A—C16—H16C	109.00
O1—C15—N2	120.20 (13)	H16B—C16—H16C	109.00
			
C8—N1—C1—Cl1	−177.53 (10)	C2—C3—C9—C8	0.9 (2)
C8—N1—C1—C2	0.9 (2)	C9—C4—C5—C6	0.4 (3)
C1—N1—C8—C7	178.16 (13)	C5—C4—C9—C3	178.26 (15)
C1—N1—C8—C9	−0.5 (2)	C5—C4—C9—C8	−0.9 (2)
C11—N2—C10—C2	97.14 (15)	C4—C5—C6—C7	1.0 (3)
C15—N2—C10—C2	−83.63 (16)	C5—C6—C7—C8	−1.7 (3)
C10—N2—C11—C12	178.31 (15)	C5—C6—C7—C16	178.65 (17)
C15—N2—C11—C12	−0.9 (2)	C6—C7—C8—N1	−177.62 (14)
C10—N2—C15—O1	2.8 (2)	C6—C7—C8—C9	1.1 (2)
C10—N2—C15—C14	−177.33 (13)	C16—C7—C8—N1	2.1 (2)
C11—N2—C15—O1	−177.96 (14)	C16—C7—C8—C9	−179.25 (14)
C11—N2—C15—C14	1.9 (2)	N1—C8—C9—C3	−0.4 (2)
Cl1—C1—C2—C3	178.03 (10)	N1—C8—C9—C4	178.84 (13)
Cl1—C1—C2—C10	−2.45 (18)	C7—C8—C9—C3	−179.02 (13)
N1—C1—C2—C3	−0.4 (2)	C7—C8—C9—C4	0.2 (2)
N1—C1—C2—C10	179.10 (14)	N2—C11—C12—C13	−0.4 (3)
C1—C2—C3—C9	−0.6 (2)	C11—C12—C13—C14	0.5 (3)
C10—C2—C3—C9	179.96 (14)	C12—C13—C14—C15	0.6 (3)
C1—C2—C10—N2	−179.27 (12)	C13—C14—C15—O1	178.08 (16)
C3—C2—C10—N2	0.2 (2)	C13—C14—C15—N2	−1.7 (2)
C2—C3—C9—C4	−178.28 (14)		

Symmetry codes: (i) *x*, −*y*+1/2, *z*−1/2; (ii) −*x*, −*y*+1, −*z*; (iii) −*x*+1, −*y*+1, −*z*; (iv) −*x*, *y*−1/2, −*z*+1/2; (v) *x*, −*y*+1/2, *z*+1/2; (vi) −*x*+1, *y*−1/2, −*z*+1/2; (vii) −*x*, *y*+1/2, −*z*+1/2; (viii) −*x*+1, *y*+1/2, −*z*+1/2; (ix) −*x*, −*y*+1, −*z*+1.

### Hydrogen-bond geometry (Å, °)

**Table:**

*D*—H···*A*	*D*—H	H···*A*	*D*···*A*	*D*—H···*A*
C3—H3···N2	0.93	2.51	2.8560 (18)	103
C11—H11···O1^vii^	0.93	2.54	3.286 (2)	137
C6—H6···Cg1^viii^	0.93	2.61	3.4457 (18)	150

Symmetry codes: (vii) −*x*, *y*+1/2, −*z*+1/2; (viii) −*x*+1, *y*+1/2, −*z*+1/2.
